# Readiness assessment as a strategy, not an afterthought: leveraging implementation science to integrate tuberculosis comorbidity care in Pakistan's health system

**DOI:** 10.3389/ftubr.2026.1809118

**Published:** 2026-04-23

**Authors:** Saima Aleem, Zohaib Khan, Bilal Ahmad Khan, Zala, Saima Afaq

**Affiliations:** 1 Institute of Public Health & Social Sciences, Khyber Medical University, Peshawar, Pakistan; 2 Office of Research Innovation and Commercialization, Khyber Medical University, Peshawar, Pakistan; 3 Department of Health Sciences, University of York, York, United Kingdom

**Keywords:** comorbidities, implementation science [MeSH], integrated care, Pakistan, readiness assessment, tuberculosis

## Abstract

Health facility readiness assessment is a foundational element in designing and implementing integrated tuberculosis (TB) and comorbidity interventions, particularly in low- and middle-income countries (LMICs) like Pakistan. The integration of TB care with management of comorbid conditions like diabetes mellitus, depression, and tobacco use is complicated and challenged by diverse, multifaceted barriers at the health system, organizational, and individual levels. Evidence-based frameworks, such as Consolidated Framework for Implementation Research (CFIR), Reach, Effectiveness, Adoption, Implementation, and Maintenance (RE-AIM) framework, the Exploration, Preparation, Implementation, Sustainment (EPIS) framework, and related tools, help researchers to systematically evaluate service system capacity for service delivery, determine operational gaps and shortcomings, and understand socio-cultural and infrastructural constraints. This ensures that subsequent interventions are not only scientifically grounded but also tailored and well-suited to local capacities, thereby enhancing the sustainability and overall effectiveness of integration. In this perspective paper, we argue that readiness assessment should be institutionalized that is, formally embedded within service delivery structure, routine policy, planning, and decision-making processes, as a strategic prerequisite for integrating tuberculosis and comorbidity care, and we highlight how implementation science frameworks can support context-sensitive planning and scale-up in Pakistan and other similar health system settings.

## Introduction

Tuberculosis (TB) remains a significant public health challenge in Pakistan, which ranks fifth among high-burden countries globally, with an estimated 510,000 persons developing TB annually, including a substantial proportion who develop drug-resistant forms of the disease (1). This burden is compounded by the fact that comorbid conditions are highly prevalent, with almost a quarter of persons with TB in Pakistan diagnosed with diabetes, and another quarter are diagnosed as pre-diabetics (2). Studies conducted in TB facilities across Peshawar documented depression in approximately 35% of persons with TB. Depression contributed to poor TB outcomes, extended hospital stays, and high costs of medical treatment (3). Secondly, the co-occurrence of tuberculosis (TB) and tobacco use, often described as ‘colliding epidemics’, poses a significant public health challenge, as tobacco smoking nearly triples the risk of both acquiring TB infection and progressing from latent infection to active disease (4).

The integration of TB services with comorbidity management represents a critical pathway for improving the health system service delivery, its efficiency, and health-related outcomes. Successful integration, however, would not only be based on clinical evidence of effectiveness; there is also a need to have a systematic knowledge of health facility readiness and implementation capacity. The Programmatic Management of Drug-Resistant Tuberculosis (PMDT) program, a nationally coordinated initiative under the Pakistan's National TB Programme (NTP) designed to diagnose, treat, and support patients with drug-resistant TB across a network of designated treatment facilities initially demonstrated strong performance; however, its progress plateaued, partly due to a predominant focus on easily measurable inputs such as infrastructure and service outputs, with comparatively limited attention to care delivery processes, provider competencies, and broader dimensions of health system readiness and implementation capacity (5). The absence of integrated and coordinated TB care systems has contributed to the emergence of multidrug-resistant TB (MDR-TB) due to diagnostic delays, delayed health-seeking behaviour, inadequate treatment regimens, weak follow-up mechanisms, and limited social support (6). In Pakistan, healthcare system fragmentation has further exacerbated these challenges, with multiple vertical, disease-specific programs such as those for tuberculosis, HIV, immunization, and non-communicable diseases operating in parallel through separate structures with limited coordination and integration (7).

Implementation science frameworks generally act as a bridge to fill the gap between evidence and practice through a systematic assessment of the contextual factors that influence intervention success. Readiness assessment is an evaluative process that systematically examines the current capabilities, availability of resources, potential barriers, and hindering factors within healthcare facilities. In the context of integrated TB-comorbidity care, readiness assessment identifies measurable material inputs, including staffing levels, infrastructure, and drug supply, as well as less quantifiable factors such as healthcare providers and staff's competencies and people-centered practices. Investigating the readiness of health facilities using an implementation science framework enables one to assess both the “hardware” (infrastructure, equipment) and the “software” (provider skills, service users’ engagement, cultural adaptation) of the healthcare system. This holistic evaluation is crucial because it informs decision-makers on whether interventions are likely to be implemented as intended and sustained over time, leveraging existing resources, if any.

In Pakistan, the considerable amount of TB-comorbidity research evidence, such as TB-tobacco (8, 9), TB-diabetes (2, 10–12), and TB-depression interventions (3, 13, 14), indicates a persistent gap between initial intervention and broader implementation. Although these studies and interventions have high technical performance and feasibility in controlled or pilot environments, their translation into routine practice and sustained scale-up remains limited. Evidence based on randomised trials, hybrid implementation studies and large-scale programme evaluation has demonstrated that this gap is not due to a lack of evidence or technical advice, but rather, a consequence of ongoing health system and implementation constraints, including staffing shortages, workflow mismatch, service delivery fragmentation, lack of coordination and electronic data fragmentation and insufficient facility level preparedness (15–17). Unless donor-funded or academic research programmes integrating TB-comorbidity care at the point of service delivery are preceded by a brief, standardised assessment of facility readiness, and adequately resourced to address identified gaps, efforts to integrate care are likely to underperform despite available guidance and intent. In this perspective, we argue that readiness assessment should be institutionalized that is, formally and sustainably embedded within routine health system structures, policy cycles, and decision-making processes, rather than treated as a one-time, research or donor funded project-specific exercise as a strategic prerequisite for integrating TB-comorbidity care in Pakistan and similar high-burden settings.

## The need to integrate TB-comorbidity care

Considering the epidemiological burden of TB comorbidities in Pakistan, an integrated care approach is imperative. The prevalence of diabetes among persons with TB screened for diabetes is significantly higher than that in the general population (18). The co-occurrence of depression among persons with TB contributes to poor treatment adherence, with studies documenting a 35% prevalence of depression in TB facilities across primary, secondary, and tertiary care settings in Peshawar (3). Tobacco use, present in approximately 20% of persons with TB, increases the risk of treatment failure, relapse, and mortality (8, 19). These comorbidities create a vicious cycle: diabetes increases the risk of TB threefold and worsens treatment outcomes (20); depression reduces treatment adherence in persons with TB and prolongs recovery (21); and tobacco use impairs immune function and leads to poor treatment outcomes (22).

In Pakistan, the fragmentation between the public and private healthcare sectors presents coordination challenges for a comprehensive TB response. Private sector providers, regarded as the first point of entry for most healthcare users, often do not follow the National TB Program (NTP) guidelines, with less than 3% of general practitioners following national guidelines for TB diagnosis and management, and 90% relying solely on chest radiography for diagnosis (23).

Public–private mix (PPM) interventions have shown promise, but implementation has been variable. Treatment success rates among PPM approaches differ markedly by facility type, with general practitioners and NGO facilities achieving the highest rates (94%–95%), private hospitals reaching 82%, and parastatal facilities losing more than half of notified TB cases to follow-up (24). This variation underscores the importance of using standardised readiness assessment frameworks that allow for context-specific interpretation, enabling identification of facility-level gaps to optimise intervention design.

## Using implementation science to inform integrated TB–comorbidity care

The complexity of TB comorbidity integration requires a systematic understanding of multilevel determinants, including outer setting factors (external policy, service users needs, resource allocation), inner setting elements (organisational culture, leadership engagement, internal communication), intervention characteristics (complexity, adaptability), and individual attributes (competence, motivation). In this context, implementation science frameworks provide structured methodologies for identifying and addressing these determinants to improve intervention outcomes (25).

Evidence from Pakistan's TB programs demonstrates that early gains achieved through well-resourced interventions were not sustained due to a narrow focus on quantifiable inputs while neglecting complex care delivery practices and psychosocial determinants (5). Similar challenges have been documented in integrated TB and diabetes care programs, where readiness assessments have revealed critical barriers, including diagnostic limitations, capacity constraints, and coordination challenges across different levels of care (26).

## Implementation science frameworks for assessing integration readiness

**Figure 1:**
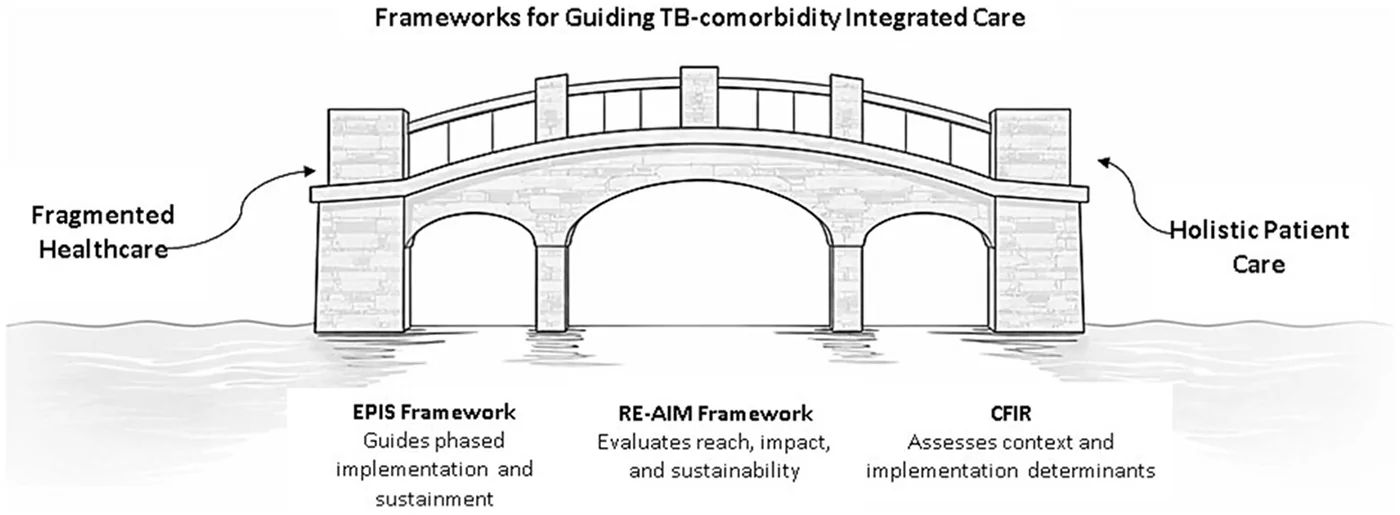
Implementation science frameworks.

In Pakistan's TB-comorbidity context, conducting a robust facility readiness assessment before implementation is not merely a preparatory step; it is the foundation upon which the success, sustainability, and cost-effectiveness of any innovation rests. Frameworks such as the Consolidated Framework for Implementation Research (CFIR) (25, 27, 28), which examines contextual determinants across multiple levels; RE-AIM, which focuses on Reach, Effectiveness, Adoption, Implementation, and Maintenance; and the Exploration, Preparation, Implementation (29), and Sustainment (EPIS) framework, which supports planning across implementation phases (30), offer structured, evidence-based pathways to ensure this foundation is strong (Figure 1).

These frameworks chart the pathway from fragmented care to holistic, equitable health outcomes. EPIS anchors the process chronologically: during the Exploration phase it surfaces unmet need and stakeholder readiness; Preparation translates that evidence into a context-sensitive implementation plan; and the Implementation and Sustainment phases ensure that the integrated TB-comorbidity service is not merely launched but embedded within routine care structures. RE-AIM operationalises success at scale by tracking whether the intervention reaches all eligible individuals equitably (Reach), produces meaningful clinical improvements (Effectiveness), is adopted by frontline providers (Adoption), is delivered with fidelity to the intended design (Implementation), and endures beyond the pilot period without continued external support (Maintenance). CFIR runs as a cross-cutting diagnostic throughout all phases, mapping the contextual determinants such as workforce capacity, leadership engagement, available resources, persons with comorbid conditions factors, and the broader policy environment, that either enable or obstruct each step of the innovation delivery. Together, the three frameworks ensure that interventions are not only scientifically sound but contextually anchored: EPIS provides the timeline, RE-AIM defines what success looks like, and CFIR explains why a given context produces the outcomes it does thereby maximising impact, safeguarding equity, and preventing resource waste in Pakistan's high-burden, resource-limited setting.

## Readiness assessment for integrated TB–comorbidity care

In Pakistan, a robust health facility readiness assessment requires a multi-method approach that incorporates both quantitative and qualitative data (26). The use of readiness heuristics such as R = MC2 (reflecting motivation, general capacity, and capacity for change) provides a structured framework to evaluate the likelihood of successful implementation (26, 31). In addition, the collaborative process incorporating a variety of stakeholders in the form of co-design workshops leads to the creation of an intervention that is highly aligned with the unique situation in the facility. These mixed-method approaches facilitate data triangulation and yield depth in understanding tangible gaps and subtle realities of the contextual influences that would otherwise not be identified.

## Why readiness matters for integrated TB–comorbidity services

The challenges of integrating TB-comorbidity care are not strictly clinical but extend into the psychosocial and infrastructural realms. The success of integrated care models is highly contingent upon the capacity of facilities to manage multiple conditions simultaneously, which in turn depends on timely and accurate diagnosis, effective referral systems, and people-centered care practices (5). Integrating depression care into TB services can significantly improve treatment adherence and reduce mortality, yet this requires facilities to have the necessary screening tools, trained personnel, and supportive infrastructures, a reality that is often lacking in LMICs (17). Similarly, the co-occurrence of TB and diabetes can lead to worse clinical outcomes if aspects such as diagnostic capacity, staffing, and supply chain management are not properly evaluated and addressed through a readiness assessment (2). By identifying these specific determinants prior to intervention design, implementers can customize strategies to enhance coordination between TB control programs and non-communicable disease management initiatives, thus ensuring a more holistic and effective response.

## Addressing health system fragmentation in TB–comorbidity care

Fragmentation of services is a common challenge in LMICs, where multiple vertical programs often operate independently with limited coordination. This phenomenon is particularly evident in settings with high burdens of TB and associated comorbidities, where disease-specific programs targeting TB, diabetes, depression, and tobacco control are frequently implemented in silos. A health facility readiness assessment grounded in implementation science frameworks provides the evidence base required to bridge these silos by mapping existing service delivery gaps, understanding referral and feedback mechanisms, and highlighting opportunities for resource sharing and capacity development (32). When integrated interventions are designed with a comprehensive understanding of these systemic weaknesses, they are better positioned to foster inter-program collaboration, streamline workflows, and create more coordinated care pathways for persons with TB and comorbid conditions (31, 33). This integrated approach is especially critical in a country like Pakistan, where limited resources and high volumes of persons with TB or diabetes necessitate efficient use of available infrastructure and human capital.

## Strengthening stakeholder engagement and ownership for integrated care

Stakeholder engagement is crucial for readiness assessment. Engaging frontline healthcare providers, facility managers, policymakers, people affected by TB and comorbidities, and community leaders in the assessment process not only provides critical insights into operational gaps but also fosters a sense of ownership over the subsequent interventions (34). Through participatory methods such as co-design workshops and stakeholder consultations, readiness assessments help to build consensus around intervention strategies and ensure that all parties are aligned in terms of goals and expectations (35). This inclusive process is integral to addressing issues of power imbalance, cultural sensitivities, and gender dynamics that may otherwise hinder implementation (36). When stakeholders are actively involved in the assessment process, they are more likely to support the design and implementation of interventions, leading to greater adherence, sustainability, and overall success of integrated programs.

## Examples of TB–comorbidity integration in Pakistan

### Integrating depression care into TB services

A successful facility readiness assessment and implementation was demonstrated by the depression care integration study in Peshawar. Three TB facilities (District Tuberculosis Office, Government City Hospital, and Khyber Teaching Hospital) underwent a systematic readiness assessment using comprehensive checklists evaluating infrastructure, human resources, organisational policies, and linkage mechanisms (3).

The readiness assessment identified multiple facility-specific gaps: the district-level facility required training of DOTS Strategy facilitators in mental health gap action program modules, the secondary care facility needed formal referral protocols with mental health services, and the tertiary care facility required service users flow management adaptations to accommodate depression screening. Targeted interventions were implemented to address these gaps before implementation.

The implementation achieved significant success: 191 of 301 persons with TB were screened for depression, with approximately 35% screening positive across varying severity levels. The systematic readiness assessment and targeted preparation enabled successful integration without disruption of routine TB care delivery (3).

### TB-Diabetes bidirectional screening

The World Diabetes Foundation's projects in Khyber Pakhtunkhwa (KP) demonstrate the success of large-scale integrated care through systematic facility strengthening. Twenty-five clinics (13 TB and 12 diabetes clinics) underwent comprehensive readiness assessments and capacity-building. The assessments evaluated infrastructure (diagnostic equipment and service users flow systems), human resources (staff training needs and role definitions), and organisational factors (management support and quality assurance mechanisms) (18).

The implementation achieved a substantial scale:12,496 persons with TB were screened for diabetes, of whom 779 were diagnosed with diabetes; 67,213 persons with diabetes were screened for TB; 49 healthcare providers were trained in integrated management; and 30 awareness campaigns were conducted. Key findings included enhanced facility readiness, workforce capacity building, ongoing supportive supervision, stakeholder engagement, and community mobilisation. Critically, the intervention influenced policy development, and bidirectional TB-diabetes screening was included in the National End TB Strategic Plan (2017–2020) for national scale-up (18).

### Tobacco cessation integration success within TB care

The TB and Tobacco Consortium's multi-country implementation study evaluated scale-up of brief tobacco cessation support within routine TB programs across Bangladesh, Nepal, and Pakistan, using four strategies: a simplified intervention, cessation training integrated into routine NTP training, tobacco indicators added to recording forms, and research embedded within TB health system. The study employed the ExpandNet nine-step scale-up framework to guide vertical and horizontal scale-up, alongside CFIR to analyse the contextual determinants of implementation. Pakistan had the largest footprint with 59 learning sites across KP Province, and achieved the study's most complete institutionalization as training was rolled out to all 121 KP facilities by 2020, the national TB patient registration and treatment record (TB01 form) was revised to include tobacco status, and cessation was formally incorporated into the 2020–2023 NTP strategic plan (15). However, Pakistan showed the study's most significant under-identification of smokers by identifying only 43% of expected prevalence which was driven by reluctance among persons with TB to disclose tobacco use, gender-concordance barriers between providers and service users, and intervention material often with adaptation or partial implementation in busy clinics.

## Discussion

Successful TB–comorbidity integration operates across three interdependent dimensions: clinical integration, which demands adequate diagnostic capacity, trained healthcare providers, and established referral mechanisms; organisational integration, which requires a supportive culture, leadership commitment, and available resources; and systems integration, which depends on policy convergence, sustainable funding, and cross-programme coordination. Readiness assessment is the mechanism through which gaps across all three dimensions are identified before implementation begins. Accumulating evidence on the TB–comorbidity integration experience in Pakistan confirms that systematic health facility readiness assessment is a major contributor to successful implementation. Its absence leads to poor results, resource waste, and sustainability failures, while its presence enables evidence-based intervention adaptation, targeted resource allocation, and enhanced stakeholder engagement.

The complexities of the Pakistan healthcare system involving federal-provincial governance, the mix of public and private service delivery, significant disease burden, and resource limitation require a sophisticated readiness assessment approach. Amalgamating the structured methodologies into implementation science frameworks helps identify systematic barriers and facilitators, facilitating the context-specific intervention designs and adaptive implementation strategies.

In Pakistan, successful TB comorbidity integration efforts, including depression care integration in Peshawar, TB-diabetes bidirectional screening in Khyber Pakhtunkhwa, and tobacco cessation scaling across multiple provinces, demonstrate the value of systematic readiness assessment in informing implementation strategies and achieving sustainable outcomes. Nevertheless, in spite of such apparent advantages, the existing body of knowledge is still limited. The majority of the studies in Pakistan are limited to small pilot tests or program-specific experiences and therefore have limited generalizability. Longitudinal monitoring of integrated care readiness over time is largely absent, limiting the evidence base from which conclusions about the sustainability and adaptability of integrated programmes can be drawn. Moreover, structural inputs are frequently recorded, but not enough focus is given to organizational culture, leadership commitment, staff motivation, and service users engagement, which are considered important to the maintenance of integrated care. Applying international implementation science frameworks such as RE-AIM and CFIR to the Pakistani context presents additional challenges. For example, limited data make it difficult to fully assess the Reach and Effectiveness aspects of RE-AIM. Similarly, the considerable variation in cultural, linguistic, and service delivery contexts across Pakistan requires these frameworks to be adapted locally, which can compromise standardization. Additionally, limited technical capacity among policymakers and program implementers may result in inconsistent or incomplete application of the framework.

Such limitations are not considered insurmountable. There are a number of possible solutions to promote readiness assessment incorporation into common practice at a faster pace. Adapting and tailoring the development of RE-AIM and CFIR instruments to the Pakistani context would make the instruments theoretically sound, applicable, and usable. Embedding implementation science training in capacity-building actions of the National TB Control Programs would enhance operational insights about these frameworks. Hybrid assessment practices complementing everyday program data with *ad hoc* facility visits, stakeholder interviews, and participatory workshops, can also produce more comprehensive, highly actionable evidence to inform readiness decisions.

## Policy implications and recommendations for integrated TB–comorbidity care

**Figure 2:**
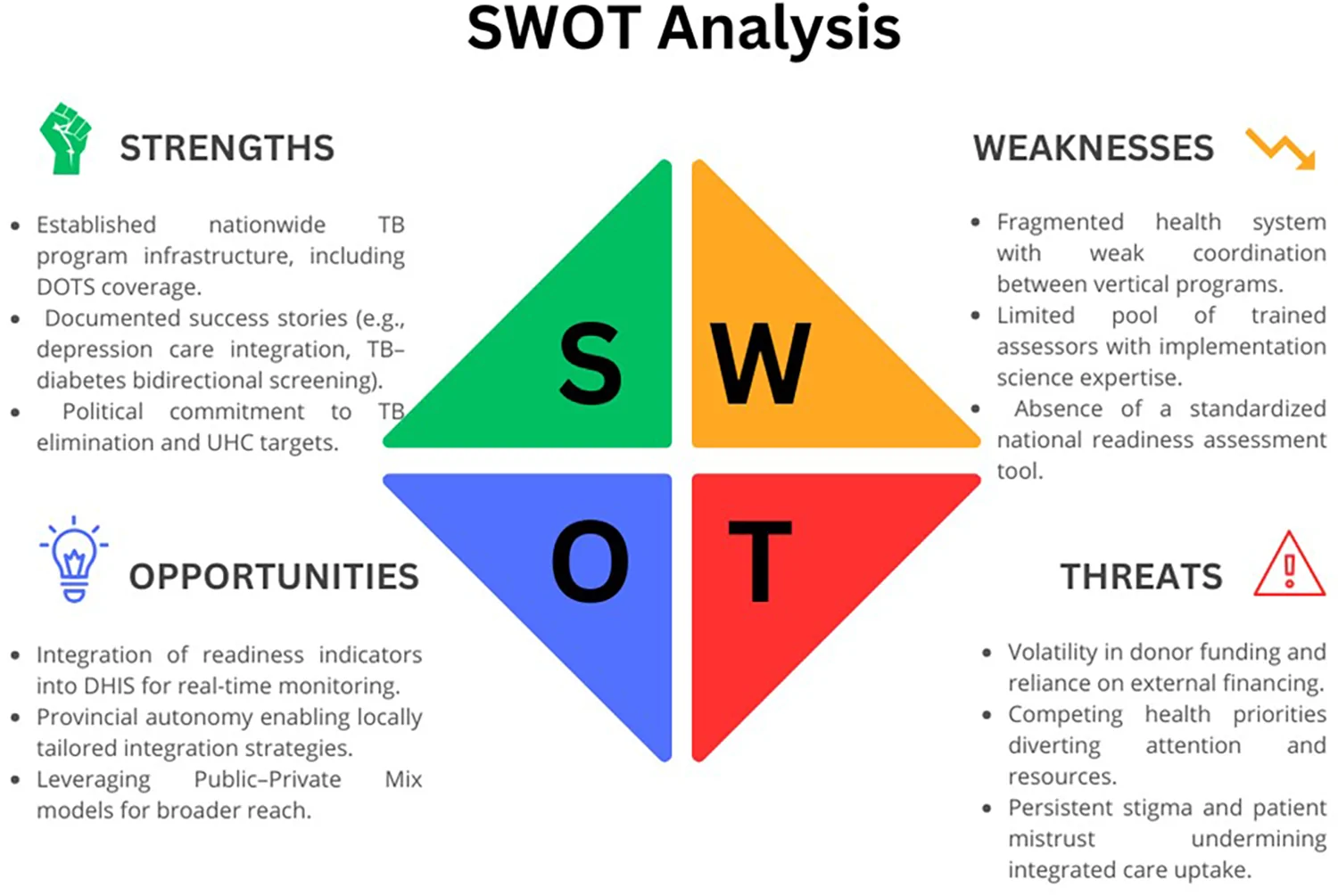
SWOT analysis of Pakistan's health system readiness for TB–comorbidity integrated care: informing evidence-based policy and implementation strategies.

Translating this evidence into actionable policy requires a systematic and realistic appraisal of Pakistan's current implementation environment. The Strengths, Weaknesses, Opportunities, and Threats (SWOT) analysis of Pakistan's TB-comorbidity system (Figure 2) reveals a mixed but workable landscape. Key strengths include the existing DOTS Strategy infrastructure which is Pakistan's nationally established network of TB treatment and supervision services, documented integration achievements in pilot settings, and political goodwill towards TB elimination and Universal Health Coverage (UHC). These are counterbalanced by notable weaknesses, particularly fragmented vertical programmes, a limited number of staff trained in integrated care delivery, and the absence of a standardised national readiness assessment tool. Externally, alignment with UHC commitments and digital health platforms such as DHIS2 present concrete opportunities, while health system fragmentation and competing programmatic priorities remain threats to sustained progress. Together, these findings provide the evidence base for the policy recommendations outlined here.

## Institutionalizing readiness assessment within national TB policy

Pakistan's commitment to TB elimination and UHC targets creates a policy window that must be used to formally mandate readiness assessment across all TB–comorbidity integration efforts. Health facility readiness assessment should be institutionalized as a prerequisite condition by the National TB Control Program, with standardized tools developed at national level and adapted to provincial contexts to ensure consistency and comparability. Incorporating readiness indicators into the National TB Strategic Plan would establish performance benchmarks and make readiness assessment a formal approval criterion rather than a discretionary research activity. This directly addresses the identified weakness of having no standardized national integrated care readiness assessment tool, while leveraging the opportunity to embed readiness indicators into the District Health Information Software 2 (DHIS2), Pakistan's national digital health data platform, for real-time monitoring at scale.

## Aligning strategic resource allocation with integration readiness

The TB-comorbidity integrated care resource allocation must be directly associated with the readiness assessment report. Investments should be made in facilities that exhibit high potential but have capacity gaps. Readiness assessments need to be embedded within program budgets to ensure they are sustainable and customary. Pakistan's PPM model presents a concrete opportunity. Because the readiness level and need vary considerably among sectors, varied allocation strategies for public, private, NGO, and parastatal facilities can maximize the reach of available resources while guarding against the threat of donor funding volatility by building assessment capacity into domestic program structures.

## Embedding readiness within system-wide integration

Integration of readiness measurements into wider health system strengthening programs such as DHIS surveillance integration, service delivery unit reviews, and facility accreditation exercises, would ensure that readiness assessment becomes a routine, system-wide function rather than a standalone activity, and can be achieved by establishing district-level readiness working groups to carry out assessments with capacity-building support on a regional basis at regular intervals. Readiness data can be employed to facilitate collaboration across programs, especially between TB, diabetes, mental health, and/or tobacco control services, to mitigate the problem of fragmentation, maximize the available resources, and create sustainable care pathways.

## Conclusion

The assessment of health facility readiness in Pakistan is the necessary foundation and prerequisite groundwork for successful TB-comorbidity integration. No matter how strong the clinical evidence or how generous the funding, integrated TB-comorbidity interventions in Pakistan will not deliver sustained results unless they are fundamentally grounded in facility readiness assessment. Policymakers, program leaders, and funders must treat readiness assessment as a mandatory prerequisite, not an optional activity, before approving or financing any integration initiative.

To operationalize this shift, Pakistan should:

1. Mandate readiness assessment in national TB-comorbidity policies, making it a formal approval criterion for new interventions.
2. Link resource allocation to readiness scores, prioritizing facilities where targeted investment will yield the highest impact.
3. Embed readiness indicators into national health information systems to enable ongoing monitoring and evaluation.
4. Develop and train dedicated district-level readiness assessment teams, ensuring capacity at the frontline.

The message is simple: **assess readiness, strengthen integration, sustain impact**. By institutionalizing readiness assessment, Pakistan can avoid repeating the costly mistakes of implementing inadequately planned interventions, protect scarce resources, and ensure that integration efforts move beyond launch toward sustained, high-fidelity delivery that improves health outcomes and advances the End TB and Universal Health Coverage goals.

## Data Availability

The original contributions presented in the study are included in the article/Supplementary Material, further inquiries can be directed to the corresponding author.
